# Prescription Department

**Published:** 1894-02

**Authors:** 


BURNS.

℞. Cocaine hydrochlorate, gr. v.

Campho-phénique,

Olive-oil, āā ℥ ss.

M. Rub up the cocaine and campho-phénique and add the olive-oil.—*Medical Review.*

NEURASTHENIA.

℞. Tr. kola.
Tr. coca, āā ℥ iss.
Citric acid, gr. xv.
Arseniate of sodium, gr. ¾.

M. Sig. Teaspoonful three times daily.—*El Siglo Medico.*

TAPE-WORM.

Dr. Duhomeau recommends:

℞. Ethereal ext. male fern, gtt. xviij.

Chloroform, gtt. l.

Castor-oil, ʒ j.

Croton-oil, gtt. i–ij

Sufficient for one treatment.—*New England Medical Monthly.*

FOR FERMENTATIVE DYSPEPSIA.

℞. Ol. creasoti puri, ♏ xij.

Spt. tenuoris, f℥ iiss.

Ammonii benzoat., ʒ ij.

Glycerin. puri, fʒ vj.

Infus. carophylli, ad. f℥ vj.

M. Sig. A tablespoonful two or three times a day, between meals, in water.—*The Asclepiad; Medical News.*

TONSILLITIS.

℞. Phenosalyl., ʒ ij.

Aq. destillat., ℥ vj.

M. Sig. Use as a gargle.—*Medical Review.*

Dr. Madan paints the throat with:

℞. Salol, ʒ j.

Camphor, gr. xv.

Glycerin, ℥ j.

Chloroform-water, ℥ iij.

Tartaric syrup, ℥ j.

When the camphor has dissolved the salol, the other substances are added.—*N. Y. Therapeutic Review.*

#### ACUTE TONSILLITIS.

D. C. Edwin Miles says, in the *Massachusett's Medical Journal:* "If the pultaceous deposits develop on the tonsils or fauces, I find no remedy to surpass the following:

℞. Ol. eucalyptus glob., ♏ xv.

Spt. camphor., ʒ iss.

Tinct. guaiac., ʒ iiiss.

Glycerin., q. s. ad. ℥ j.

M. Sig. Put 10 drops on a bit of sugar, and allow it to dissolve in the mouth, every hour or two.

#### OBESITY.

℞. Phytoline (Walker), fℨ j.
Tinct. digitalis, ℈ j.
Fl. ext. fuscus vesiculosis, fℨ j.

M. Sig. Twenty drops before and after the three daily meals.—*Medical Review.*

#### WARTS.

℞. Salicylic acid, gr. xv.
Lactic acid, gr. xv.
Collodion, ʒ ss.

M. Sig. Apply twice daily. — *Deutsche med. Zeitung.*

#### OVARITIS.

M. Winternitz prescribes a teaspoonful of the following mixture at bedtime in a glass of water:

℞. Sulphate of soda, ʒ iv.

Sulphur, ʒ j.

Sugar, ʒ v.

Essence of peppermint, q. s.

The abdomen is rubbed with the oitment here mentioned:

℞. Ichthyol.

Lanolin, āā p. æq.

The patient is kept in bed and warm vaginal injections are used.—*Medical and Surgical Reporter.*

#### WHOOPING-COUGH.

Prof. C. Sée prescribes:

℞. Pow'd belladonna-root, gr. ⅙.
Dover's powder, gr. ss.
Sublimed sulphur, gr. viij.
White sugar, gr. viij.

M. Sig. For one dose from two to ten times daily, according to the age of the patient and the effect produced.

